# Baseline Cell-Free DNA Can Predict Malignancy of Nodules Observed in the ITALUNG Screening Trial

**DOI:** 10.3390/cancers16122276

**Published:** 2024-06-19

**Authors:** Simonetta Bisanzi, Donella Puliti, Giulia Picozzi, Chiara Romei, Francesco Pistelli, Annalisa Deliperi, Giulia Carreras, Giovanna Masala, Giuseppe Gorini, Marco Zappa, Cristina Sani, Laura Carrozzi, Eugenio Paci, Rudolf Kaaks, Francesca Maria Carozzi, Mario Mascalchi

**Affiliations:** 1 Institute for Cancer Research, Prevention and Clinical Network (ISPRO), 50139 Florence, Italy; s.bisanzi@ispro.toscana.it (S.B.); g.picozzi@ispro.toscana.it (G.P.); g.carreras@ispro.toscana.it (G.C.); g.masala@ispro.toscana.it (G.M.); g.gorini@ispro.toscana.it (G.G.); m.zappa@ispro.toscana.it (M.Z.); c.sani@ispro.toscana.it (C.S.); paci.eugenio@gmail.com (E.P.); frakaro@gmail.com (F.M.C.); 2 Division of Radiology, Cisanello Hospital, Azienda Ospedaliera Pisana, 56124 Pisa, Italy; ch.romei@ao-pisa.toscana.it (C.R.); a.deliperi@ao-pisa.toscana.it (A.D.); 3 Department of Surgical, Medical and Molecular Pathology and Critical Care Medicine, University of Pisa, 56126 Pisa, Italy, laura.carrozzi@unipi.it (L.C.); 4 Pulmonary Unit, Cardiothoracic and Vascular Department, Pisa University Hospital, 56124 Pisa, Italy; 5 Division of Cancer Epidemiology (C020), German Cancer Research Center (DKFZ), Im Neuenheimer Feld 280, 69120 Heidelberg, Germany; r.kaaks@dkfz-heidelberg.de (R.K.); mario.mascalchi@unifi.it (M.M.); 6 Translational Lung Research Center Heidelberg (TLRC-H), German Center for Lung Research (DZL), 69120 Heidelberg, Germany; 7 Department of Clinical and Experimental Biomedical Sciences “Mario Serio”, University of Florence, 50121 Florence, Italy

**Keywords:** biomarkers, cell-free DNA, low-dose CT, lung cancer, prediction, screening

## Abstract

**Simple Summary:**

We investigated whether the baseline plasma cell-free DNA might help to differentiate malignant nodules from benign lesions observed in screening low-dose computed tomography (LDCT) examinations. Plasma cell-free DNA was determined before the first LDCT in 137 participants in the ITALUNG trial, including 29 with screen-detected malignant nodules (17 prevalent and 12 incident) and 108 with benign nodules. In subjects with prevalent lung cancers (LC), the radiological characteristics well differentiated the malignant nodule, and the cell-free DNA was markedly increased. A total of 75% of subjects with incident LC showed a baseline cell-free DNA ≥ 3.15 ng/mL, compared to 34% of subjects with benign nodules (*p* = 0.006). Moreover, the cell-free DNA correlated (*p* = 0.001) with the tumor growth measured with nodular volume doubling time. The baseline plasma cell-free DNA is an independent, potentially useful biomarker in the LC screening process and should be further investigated.

**Abstract:**

The role of total plasma cell-free DNA (cfDNA) in lung cancer (LC) screening with low-dose computed tomography (LDCT) is uncertain. We hypothesized that cfDNA could support differentiation between malignant and benign nodules observed in LDCT. The baseline cfDNA was measured in 137 subjects of the ITALUNG trial, including 29 subjects with screen-detected LC (17 prevalent and 12 incident) and 108 subjects with benign nodules. The predictive capability of baseline cfDNA to differentiate malignant and benign nodules was compared to that of Lung-RADS classification and Brock score at initial LDCT (iLDCT). Subjects with prevalent LC showed both well-discriminating radiological characteristics of the malignant nodule (16 of 17 were classified as Lung-RADS 4) and markedly increased cfDNA (mean 18.8 ng/mL). The mean diameters and Brock scores of malignant nodules at iLDCT in subjects who were diagnosed with incident LC were not different from those of benign nodules. However, 75% (9/12) of subjects with incident LC showed a baseline cfDNA ≥ 3.15 ng/mL, compared to 34% (37/108) of subjects with benign nodules (*p* = 0.006). Moreover, baseline cfDNA was correlated (*p* = 0.001) with tumor growth, measured with volume doubling time. In conclusion, increased baseline cfDNA may help to differentiate subjects with malignant and benign nodules at LDCT.

## 1. Introduction

The term cell-free DNA (cfDNA) refers to the total amount of fragmented DNA in plasma or serum, which can be derived from multiple sources, including tumor cells, surrounding non-neoplastic epithelial cells, and leukocytes, particularly neutrophils [1,2,3,4,5]. cfDNA is an established biomarker of the presence and aggressiveness of lung cancer (LC) [6], and along with other biomarkers present in the blood, overall allowing a “liquid biopsy”, is beginning to influence cancer patients’ prognosis and treatment [2,6,7,8]. So far, cfDNA has been evaluated for use in LC screening studies with two aims: (1) to predict the risk of developing LC in recruited smokers and former smokers before chest low-dose computed tomography (LDCT) [9,10,11] and (2) to predict malignancy of nodules detected in chest LDCT [10,11]. In particular, LDCT shows a great number of nodules in subjects screened for LC, of which just a small minority correspond to LC [12]. For this reason, in the last few years, risk stratification of nodules has been implemented based on nodule features derived from LDCT, according to the Lung-RADS 1.1 or Lung-RADS v2022 classifications [13,14,15], alone or combined with other individual factors with the computation of risk scores as proposed by the group at Brock University (Canada) [16,17,18]. 

We formerly analyzed in blood samples obtained before initial LDCT (iLDCT) the performance of cfDNA quantification, loss of heterozygosity, and microsatellite instability in differentiating subjects with and without LC (including those with negative LDCT) in the ITALUNG trial [11], with promising results. In the present study, we re-analyzed a subset of the data, integrating them with information on nodule characteristics obtained by a review of the iLDCT images, in order to evaluate if the baseline cfDNA could support discriminating between benign and malignant lung nodules. In particular, we considered here separately subjects with prevalent and incident LC and compared the predictive value of baseline cfDNA with those of LDCT features alone and of the Brock score. Moreover, assuming a correlation between cfDNA concentration and speed of tumor growth [1,2,3,4,5], we explored whether cfDNA might be inversely correlated with volume doubling time (VDT) measured in serial LDCT in screen-diagnosed LC [19]. 

The results indicate that the radiological characteristics successfully differentiated malignant and benign nodules in subjects with prevalent LC, in whom the cell-free DNA was markedly increased. Differently, the radiological characteristics failed to differentiate between malignant and benign nodules in subjects with incident LC, who, however, showed a baseline cell-free DNA ≥ 3.15 ng/mL more frequently than subjects with benign nodules. Moreover, the cell-free DNA correlated with the tumor growth measured with nodular volume doubling time. 

## 2. Materials and Methods

### 2.1. Participants’ Selection

The protocols, LDCT results, and mortality data of the ITALUNG trial (Clinical Trial Registration number NCT02777996) were previously reported [20,21,22]. Eligible were subjects 55–69 years of age with a smoking history > 20 pack-years and who were current smokers or had quit in the last 10 years. Between 2004 and 2006, 1613 subjects were randomized to receive 4 annual LDCTs, and 1593 subjects received usual care. The iLDCT was obtained in 1406 subjects of the active arm. In 1356 of the 1406 subjects of the active arm, a blood sample was taken at enrolment [10] (the baseline sample) on average 1 month before LDCT and was immediately processed under specific operating procedures and stored in the ITALUNG biobank. In ITALUNG, the presence of a non-calcified solid nodule ≥ 5 mm in mean diameter or a non-solid nodule ≥10 mm in mean diameter or the presence of a part-solid nodule qualified test positivity at iLDCT [20], whereas a new appearance of a solid or part-solid nodule of at least 3 mm in mean diameter, as well as growth of at least 1 mm in mean diameter of a pre-existing solid or part-solid nodule, qualified positivity at the next annual LDCT examinations [21]. 

From the data collected for the previous study [11], we selected samples with a baseline cfDNA quantification from subjects with screen-detected LC (29/36 = 81%) or subjects with positive iLDCT but whose nodules did not evolve in LC (i.e., benign nodules) (110/128 = 86%). The iLDCT images could not be retrieved from two subjects with benign nodules who were excluded from the present analysis. Seventeen prevalent LCs were diagnosed at baseline iLDCT, and 12 incident LCs were diagnosed in the 2nd–4th screening rounds (1 at the second, 5 at the third, and 6 at the fourth). For two subjects with multiple LC (one with 1 adenocarcinoma and 1 small cell lung cancer-limited disease, and another with 2 adenocarcinomas and 1 histologically undefined LC), we considered the more advanced cancer. 

### 2.2. Plasma DNA Quantification 

The amount of plasma cfDNA was quantified some years ago in a previous study [11] using real-time quantitative polymerase chain reaction (rtPCR) amplification of the human telomerase reverse transcriptase gene (hTERT), a single copy gene mapped on 5p15.33, as previously detailed [10].

Blood was collected in a K3-EDTA-containing tube and processed within an hour after the blood sample withdrawal. Plasma was separated from the cellular fraction by centrifuging at 900× *g* at 4 °C for 10 min and freezing at −80 °C. DNA was isolated from 1 mL of plasma sample by using a standard method based on affinity purification (Qiagen), according to the blood and body fluids protocol. The sequences of the primers and the probe, designed to specifically amplify hTERT, were the following: primer forward, 5′-GGC ACA CGT GGC TTT TCG-3′; primer reverse, 5′-GGT GAA CCT CGT AAG TTT ATG CAA-3′; probe, VIC 5′-TCA GGA CGT CGA GTG GAC ACG GTG-3′ TAMRA. The amplicon size was 98 bp. Amplifications were carried out in a 7500 Real-Time PCR System (Life Tecnologies, Carlsbad, CA, USA). Linear amplification down to the last point dilution representing 10 pg of target DNA was obtained in each experiment (correlation coefficient, 0.999–0.995; slope, 3.25–3.35). All data were analyzed using the 7500 Real-Time PCR System Sequence Detection software v.1.2.3 to obtain the absolute amount of cfDNA in the sample.

The value of cfDNA was expressed as ng of DNA per ml of a plasma sample from which DNA was isolated. 

### 2.3. LDCT Assessment

A sometimes-neglected aspect of LDCT screening practice is that an LC can often be retrospectively tracked to a pre-existing smaller lung nodule that, because of its size, has escaped detection or report [23,24]. However, the inclusion of such cases allows a more comprehensive evaluation of the potential of cfDNA, especially baseline cfDNA, as a biomarker for LC screening. 

Two radiologists with more than 15 years of experience in LC screening (MM and GP) reviewed in consensus the iLDCT of the 29 subjects with screen-detected LC and of the 108 subjects with benign nodules. One of them (MM) measured the mean diameter of lung nodules in the iLDCT, classified them according to the Lung-RADS 1.1 criteria [13,14], and extracted the LDCT information that, along with age, gender, and familial history of LC collected from the enrolment questionnaire, is necessary to compute the Brock score. The latter include the size (mean diameter) of the nodule, its density (solid, non-solid, mixed), the location of the nodule in the upper lobes of the lung, the number of nodules, the presence of pulmonary emphysema, and nodule spiculations [16]. A 5% threshold of the Brock score was chosen to suspect nodule malignancy [16,17,18].

Finally, in 14 subjects with screen-detected LC (4 prevalent and 10 incident LC) appearing as solid nodules and having multiple LDCT before diagnosis, we could measure VDT from lesion maximum and perpendicular diameters according to Hasegawa et al. [19]. In cases with more than two serial LDCT examinations, the first and the last were considered. 

### 2.4. Statistical Analyses

Descriptive statistics were reported for the entire dataset (benign nodules and LC by round at diagnosis). Differences between proportions were tested using chi-square tests. The correlation between cfDNA level and VDT was measured using the Pearson coefficient correlation (ρ) and a multivariate linear regression model adjusted for the size of the nodule.

Analysis of the cfDNA threshold value that best discriminated subjects with malignant and subjects with benign nodules was performed using the Youden method [25], which defines the optimal cut-off as the point that maximizes the sum of sensitivity and specificity.

We measured the area under the receiver operator curve (AUROC) for malignancy prediction of the plasma cfDNA, size of the nodule, Lung-RADS 1.1 class, and Brock score. 

Optimistic estimation of the AUROC due to the overfitting of statistical models is a frequent problem in small datasets like ours. To overcome this, we corrected all the estimates of the AUROC for over-optimism using cross-validation with a replication approach [26] that is less biased than traditional approaches for this purpose [26]. Accordingly, the dataset was randomly split into two separate, equally sized groups in order to ensure that each was representative of the underlying population. The predictive model was fitted to the first dataset. The resulting model was then applied to the second dataset, and the AUROC was calculated. This process was repeated 100 times. The resulting 100 AUROC estimates were averaged to produce a single optimism-corrected estimate of the AUROC, and the 2.5 and 97.5 percentiles are presented as 95% uncertainty interval limits. 

All analyses were performed using Stata version 16.1.

## 3. Results

**Figure 1:**
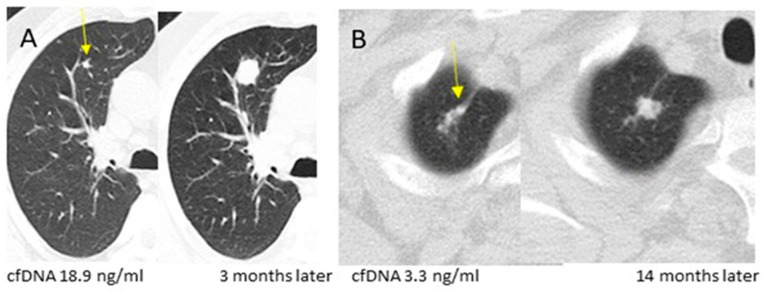
Increased baseline cfDNA in two subjects with prevalent (**A**) and incident (**B**) screen-detected lung cancer appearing as Lung-RADS 2 (<6 mm in diameter) solid nodules at iLDCT. (**A**) Prevalent adenosquamous LC in a subject with baseline plasma cfDNA of 18.9 ng DNA/mL appearing at iLDCT as a solid nodule of 5.9 mm in diameter in the anterior right lobe (arrow left panel); the nodule showed remarkable growth at 3 months follow-up LDCT (right panel). The Brock score at iLDCT was 0.1%. (**B**) Incident adenocarcinoma in a subject with baseline plasma cfDNA of 3.3 ng DNA/mL appearing at iLDCT as a solid nodule of 4.6 mm in diameter within a scar in the right apex (arrow left panel); the nodule showed growth in a follow-up LDCT obtained 14 months later (right panel). The Brock score was 0.4% at the iLDCT and 6.9% at the last LDCT.

Table 1 reports the distribution of demographic and historical characteristics, initial LDCT findings and Brock score, and results of cfDNA (ng DNA/mL plasma) in the 108 subjects with benign nodules and in the 29 (17 prevalent and 12 incident) subjects (Figure 1) with screen-detected LC. There was no statistically significant difference in age, gender, smoking status, familial history of LC, or frequency of emphysema in iLDCT. Subjects with screen-diagnosed LC had significantly higher smoking exposure as compared to subjects with benign nodules (median pack-years: 52 vs. 40.5, *p* = 0.004). Subjects with prevalent LC showed different characteristics from those with incident LC. In particular, almost half (8/17 = 47%) of the subjects with prevalent LC had more than one nodule or had at least one nodule with spiculated borders at iLDCT, whereas the presence of spiculation was not observed (0%) in subjects with incident LC and was rare (2.8%) in subjects with benign nodules. In addition, 94% (16/17) of the LC diagnosed at the 1st round appeared as large lesions, which were classified as Lung-RADS 4, whereas only 19% (21/108) of benign nodules and 8% (1/12) of incident LC were in the category Lung-RADS 4 (*p* < 0.001). The best discriminating threshold of cfDNA between all subjects with LC and the subjects with benign nodules was 3.15 ng DNA/mL plasma (sensitivity 79%, specificity 66%).

Notably, 10 of the 12 (83%) incident LC were already visible at iLDCT (Figure 1), even if their mean diameters and Brock scores were not different from those of benign nodules (7.2 mm vs. 6.9 mm, *p* = 0.88; 4.2% vs. 3.2%, *p* = 0.62). LC appeared as a new nodule at the 3rd annual LDCT rounds in the two other subjects with incident LC with no corresponding nodule (Lung-RADS 1) at iLDCT. However, 75% (9/12) of subjects with incident LC showed a baseline cfDNA ≥ 3.15 ng/mL (including 7 subjects with small nodules that were classified as Lung-RADS 2 or 3 and 2 subjects without any nodule at iLDCT) compared to 34% (37/108) subjects with benign nodules (*p* = 0.006). Excluding LC diagnosed at the 1st round, the probability of having an LC diagnosed at the 2nd, 3rd, or 4th screening rounds was significantly (*p* = 0.006) higher for subjects with an increased baseline cfDNA (>3.15 ng/mL) (9/46 = 20%) compared to those with a baseline cfDNA below the threshold (3/74 = 4%) (see Table 1). 

Table 2 shows over-optimism-corrected diagnostic performance (AUROC, 95% uncertainty interval limits) of cfDNA, size of the main nodule, Lung-RADS, and Brock score of the nodule at iLDCT for differentiation between LC and benign nodules by lag-time (interval between date of sample and date of diagnosis).

The AUROC was similar for all four variables (range: 0.72–0.76) when all screen-diagnosed LC were considered. The Brock score yielded an AUROC of 0.91 (0.75–0.99) for the prevalent LC but only 0.44 (0.26–0.65) for the incident LC. Notably, the cfDNA was the only parameter maintaining some albeit low ability (AUROC = 0.61; 95%CI 0.20–0.85) in distinguishing between incident LC and benign nodules at iLDCT.

In our dataset, the cfDNA was not significantly associated with the number of nodules (*p* = 0.266), the initial size of the nodule (*p* = 0.093), the Lung-RADS (*p* = 0.128), or the Brock score (*p* = 0.223). The proportion of lung cancer cases with cfDNA ≥ 3.15 does not differ by histotype (14/18 = 78% for adenocarcinoma and 9/11 = 82% for other histology categories, *p* = 0.794). 

Table 3 shows the number of benign nodules, the number of LC, and the negative and positive predictive values (NPV, PPV) based on the 3.15 ng/mL cfDNA threshold stratified by size, Lung-RADS category, and Brock score.

It is noteworthy that among nodules smaller than 8 mm, the proportion of malignant nodules is 2% (1/53) in those with cfDNA below the threshold and 21% (8/38) in those with cfDNA above the threshold (*p* = 0.0025). Among nodules belonging to Lung-RADS 1/2, the proportion of malignant nodules is 4% (2/47) in those with cfDNA below the threshold and 24% (8/33) in those with cfDNA above the threshold (*p* = 0.0081). Finally, among nodules with a Brock score <5%, the proportion of malignant nodules is 3% (2/60) in those with cfDNA below the threshold and 19% (7/37) in those with cfDNA above the threshold (*p* = 0.010). In addition, 92% (22/24) of nodules in the Lung-RADS 3 or 4A categories with cfDNA < 3.15 were found to be benign nodules at subsequent follow-up LDTC. 

Remarkably, when we used the ITALUNG biomarker panel (IBP) with the 5 ng/mL plasma threshold for cfDNA in our dataset of subjects with a nodule, the latter was positive in 42% (45/108) of benign nodules, 100% (17/17) of LC screen-detected at the first round, and 67% (8/12) of screen-detected incident LC with a not significant (*p* = 0.098) difference between benign nodules and incident LC. However, when we adopted the 3.15 ng/mL threshold for cfDNA, the IBP was positive in 49% (53/108) of benign nodules, 100% (17/17) of LC screen-detected at the first round, and 83% (10/12) of screen-detected incident LC. The difference between benign nodules and screen-detected incident LC was significant (*p* = 0.024). Overall, the AUROC of IBP with the 3.15 ng/mL threshold was similar to the AUROC of the only cfDNA (0.72 vs. 0.76, *p* = 0.499).

**Figure 2:**
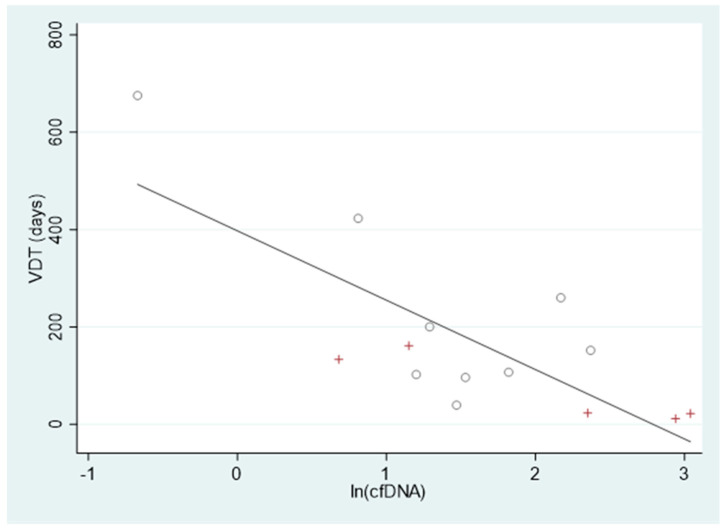
Correlation between cfDNA and tumor volume doubling time. The natural logarithm of baseline cfDNA (ng/mL) (x-axis) and tumor volume doubling time (days) (y-axis) shows an inverse significant correlation (ρ = −0.77; 95% CI: −0.92 to −0.40) in 14 subjects with screen-detected (4 prevalent and 10 incident LC) (○ = adenocarcinomas; + = others lung cancers, including 2 squamous carcinomas, 1 adeno-squamous carcinoma, 1 pleomorphic carcinoma, and 1 small cell carcinoma).

The mean VDT of the 14 screen-detected LC was 172 days, with a non-significant longer VDT for the adenocarcinomas (228.5 vs. 70.5 days; *p* = 0.12). The baseline cfDNA was inversely correlated with tumor VDT (ρ = −0.77; 95% CI: −0.92 to −0.40) (Figure 2) but not with the size of the malignant nodule at first LDCT screening (ρ = −0.23; 95% CI: −0.68 to 0.35). 

The multivariate linear regression model confirms the association of cfDNA with VDT (*p* = 0.001).

## 4. Discussion

The role of cfDNA in early LC detection and, hence, in screening is uncertain [2,6]. Two case–control studies reported that the amount of plasma cfDNA was increased in patients with clinically presenting and variably advanced LC when compared with healthy controls [27,28]. A study conducted in the Italian MILD trial of LC screening indicated that cfDNA collected before iLDCT was not a sensitive biomarker with respect to the diagnosis of LC during the next five-year period of the trial [9]. In the same study, the cfDNA was associated with more advanced disease stages and a poorer prognosis; moreover, serial sampling revealed that plasma cfDNA increased approaching LC diagnosis [9]. However, in such a study, the radiological features of the LC or the individual risk profile were not evaluated. The performance of cfDNA quantification was also evaluated in two prior investigations by our group in the ITALUNG study [10,11] as part of a panel of biomarkers called IBP, including analysis of genomic instability in blood and sputum. In particular, baseline cfDNA using a cut-off ≥ 5 ng DNA/mL plasma was increased in 18% of 235 subjects with negative iLDCT, in 14% of 128 subjects with positive iLDCT (no LC), in 66.7% of 18 subjects with LC diagnosed at baseline LDCT, and in 29.4% of 18 subjects with LC diagnosed at annual repeat LDCT [11]. 

In the present investigation, we evaluated cfDNA not as a biomarker for LC screening in general, but as a biomarker to anticipate malignancy of lung nodules detected at iLDCT. The detailed performance of the complete IBP for the latter purpose was not fully reported here because it did not increase the predictive capability of cfDNA alone to differentiate incident LC and benign nodules. The choice to select cfDNA alone to differentiate malignant and benign lung nodules detected by LDCT appears reasonable in light of the cfDNA physiopathology assumed in cancer subjects that comprises several mechanisms of fragmented DNA release from alive or dead tumor cells, including apoptosis, necrosis, production of neutrophil extracellular traps and extracellular vesicles, and pyroptosis (caspase 1-dependent cell death) [1,2,3], but also from leukocytes [4,5]. It is also in line with the results of cfDNA obtained in patients with various cancer types [29]. As a second novelty as compared to the prior investigations in the ITALUNG study [10,11], we applied here to malignant and benign nodules observed at iLDCT the Lung-RADS 1.1 classification and the Brock score, which are both capable of anticipating malignancy of a given nodule, especially when detected at prevalent screening, and are now recommended in screening practice [13,15,18]. By reviewing the iLDCT of all screen-detected LC in ITALUNG, we could observe that 10 of the 12 incident LC were already visible at iLDCT (7 classified as Lung-RADS 2, 2 as Lung-RADS 3, and 1 as Lung-RADS 4A), and only in two subjects did LC appear as a new nodule at the 3rd annual LDCT round. 

The main result of the present investigation is that the quantification of cfDNA could be more advantageously used when applied to subjects with a positive iLDCT in order to better distinguish malignant and benign nodules, thus having a prognostic value. In this specific setting, the optimal threshold could be lower (3.15 ng DNA/mL plasma) than the threshold of 5 ng DNA/mL plasma that differentiated subjects with LC from all the remainder of subjects, including those with negative LDCT [10]. Moreover, we observed that the AUROC of the cfDNA, nodule size, Lung-RADS classification, and Brock score at iLDCT were similar (range 0.72–0.76) and good for prevalent LC. In particular, the over-optimism-corrected predictive value of the Brock score for malignant nodules diagnosed within 1 year of iLDCT was already very high (0.91). Differently, when we considered only screen-detected incident LC, the discriminating value of all the predictors dropped (from a 0.82–0.91 to a 0.44–0.61 range). Notably, only cfDNA maintained a potential predictive value for having an LC diagnosed in the subsequent rounds. Furthermore, our results suggest that cfDNA may help to discriminate between benign and malignant nodules even within the same size class, Lung-RADS category, or Brock score class. Indeed, 92% of nodules that were Lung-RADS 3 or 4A and had cfDNA < 3.15 ng/mL were found to be benign nodules at subsequent follow-up LDCT. Nodules in Lung-RADS categories 3A and 4 represent indeterminate nodules for which LDCT follow-up at 3 or 6 months, but no immediate investigation, is recommended. The cfDNA value could then be used to monitor this category of nodules over time or to provide differentiated follow-up guidance. 

Finally, the inverse correlation between cfDNA and VDT that we observed in 14 LC cases extends to the small-size LC detected in the screening setting the notion that plasma cfDNA is increased in tumours with a higher growth rate that is translated in a shorter VDT [3,12]. Despite the small sample size, this observation suggests a complementary role of cfDNA and LDCT radiologic characteristics in the management of suspicious nodules discovered in screening that deserves further investigation. 

We recognize the following limitations of the present study. First, we evaluated a single cohort with a small sample size, albeit in a well-controlled randomized trial. Therefore, our results (including the choice of the optimal cut-off) need to be replicated and validated in independent samples from other LC screening studies and from healthy, non-smoking subjects. This is planned to be conducted prospectively in the context of the ongoing CCM/Italung2 study [15], in which a baseline blood sample was obtained from individuals undergoing LDCT screening. Second, the small sample size precluded matching subjects with malignant and benign nodules for potential confounders, including pack years, number of nodules, nodule size, Lung-RADS, and Brock score. However, we corrected for over-optimism inherent to small datasets by applying cross-validation with a replication approach [26]. Third, concerns about standardization and technical validation of cfDNA have been raised [2,7]. However, in the IBP, we set up rigorous pre-analytical and analytical conditions to ensure the integrity of the samples and high-quality molecular tests [10]. Fourth, we assessed the non-specific total cfDNA associated with screen-diagnosed LC, whereas methods are now available and increasingly implemented for the identification of free DNA originating from LC, so-called tumor circulating DNA (ctDNA). However, ctDNA usually represents a small (0.01% to 10%) fraction of the total plasma cfDNA, requires costly technologies, and ctDNA shedding from different histotypes of LC is heterogeneous, with adenocarcinomas that generally shed significantly less ctDNA than squamous cell carcinomas [30,31]. Possibly, in a screening setting, a more sensitive but less specific biomarker such as cfDNA may be preferable until a variegated and less expensive panel for LC tumor-specific biomarkers is available. 

## 5. Conclusions

Our study indicates that increased plasma cfDNA at baseline may help to differentiate subjects with benign and malignant nodules at iLDCT and to support/anticipate malignancy suspicion of slowly growing nodules that take years to reach the size, Lung-RADS category, or Brock score threshold that justify diagnostic work-up. These findings and the inverse correlation of baseline cfDNA and VDT in screen-diagnosed LC suggest that a multimodal screening approach integrating non-invasive biomarkers and imaging techniques might improve the screening algorithm.

## Figures and Tables

**Table 1:** Demographic, individual smoking history, familial history of LC, initial LDCT findings and Brock score, and results of cfDNA (ng DNA/mL plasma) in the 108 subjects with benign nodules and 29 screen-detected LC included in the study.

	Benign Nodules (*n* = 108)	LC Screen-Diagnosed at 1st Round (*n* = 17)—Prevalent LC	LC Screen-Diagnosed at 2nd–4th Round (*n* = 12)—Incident LC	*p*-Value
**Demographics, individual smoking history, and familial history of LC**				
Gender: *n* (% female)	42 (39%)	4 (24%)	2 (17%)	0.176
Age at randomization (mean)	61.0	62.9	63.0	0.1136
Age at randomisation: *n* (%)				
<60 years	47 (44%)	4 (24%)	4 (33%)	
60–64 years	31 (29%)	6 (35%)	1 (8%)	
65–70 years	30 (28%)	7 (41%)	7 (58%)	0.123
Smoking status *n* (%)				
Former	30 (28%)	4 (24%)	3 (25%)	
Current	78 (72%)	13 (76%)	9 (75%)	0.922
Pack-years (median)	40.5	52.5	50.7	0.015
Pack-years class: *n* (%)				
[20–30) pack-years	21 (19%)	0 (0%)	1 (8%)	
[30–40) pack-years	28 (26%)	4 (24%)	3 (25%)	
[40–50) pack-years	33 (31%)	3 (18%)	1 (8%)	
≥50 pack-years	26 (24%)	10 (59%)	7 (58%)	0.018
Family history of LC: *n*, (% yes)	13 (12%)	3 (18%)	1 (8%)	0.731
Body Max Index* (mean)	25.6	27.1	24.7	0.427
Body Max Index*: *n* (%)				
[18–25)	49 (46%)	5 (29%)	6 (50%)	
[25–30)	50 (47%)	8 (47%)	5 (42%)	
≥30	8 (7%)	4 (24%)	1 (8%)	0.284
Asbestos exposure: *n* (% yes)	8 (7%)	1 (6%)	1 (8%)	0.965
Silica exposure: *n* (% yes)	3 (3%)	0 (0%)	0 (0%)	0.662
Chemicals (solvents, detergents, etc.) exposure: *n* (% yes)	33 (31%)	8 (47%)	2 (17%)	0.204
**Initial LDCT findings**				
Emphysema: *n* (% yes)	38 (35%)	7 (41%)	4 (33%)	0.877
Number of nodules (median)	1	1	1	0.658
Number of nodules: *n* (%)				
0	0 (0%)	0 (0%)	2 (17%)	
1	69 (64%)	9 (53%)	6 (50%)	
2	24 (22%)	3 (18%)	2 (17%)	
≥3	15 (14%)	5 (29%)	2 (17%)	0.001
Spiculation: *n* (% yes)	3 (2.8%)	8 (47.1%)	0 (0%)	<0.001
Size (mm) of main nodule (mean)	6.9	22.8	7.2	<0.001
Size (mm) of main nodule: *n* (%)				
<6 mm	60 (56%)	1 (6%)	5 (42%)	
≥6 to <8 mm	22 (20%)	1 (6%)	2 (17%)	
≥8 to <15 mm	21 (19%)	6 (35%)	2 (17%)	
≥15 mm	5 (5%)	9 (53%)	3 (25%)	<0.001
Lung-RADS (version 1.1): *n* (%)				
1	0 (0%)	0 (0%)	2 (17%)	
2	70 (65%)	1 (6%)	7 (58%)	
3	17 (16%)	0 (0%)	2 (17%)	
4A	16 (15%)	3 (18%)	1 (8%)	
4B	3 (3%)	3 (18%)	0 (0%)	
4X	2 (2%)	10 (59%)	0 (0%)	<0.001
**Brock score (%) at initial LDCT**				
mean	3.2%	29.7%	4.2%	<0.001
category: *n* (%)				
<5%	88 (81%)	1 (6%)	8 (67%)	
≥5%	20 (19%)	16 (94%)	4 (33%)	<0.001
**cfDNA (ngDNA/mL plasma)**				
mean	3.3	18.8	4.8	<0.001
category: *n* (%)				
<3.15	71 (65.7%)	3 (17.7%)	3 (25.0%)	
[3.15–5)	21 (19.4%)	2 (11.8%)	5 (41.7%)	
≥5	16 (14.8%)	12 (70.6%)	4 (33.3%)	<0.001

**Table 2:** Over-optimism corrected area under the receiver operator curve (95% uncertainty interval limits) of cfDNA, size of the main nodule, Lung-RADS, and Brock score of the nodule at iLDCT for differentiation between lung cancer and benign nodules by lag-time.

	All Screen-Diagnosed LC vs. Benign Nodules	Screen-Diagnosed LC at 1st Round vs. Benign Nodules	Screen-Diagnosed LC at 2nd–4th Rounds vs. Benign Nodules
Lag-time (months) *: min–max	0–75 months	0–7 months	18–75 months
cfDNA (ng DNA/mL plasma)	0.75 (0.62–0.83)	0.82 (0.66–0.95)	0.61 (0.20–0.85)
Size of the main nodule (mm)	0.72 (0.60–0.84)	0.89 (0.75–0.99)	0.42 (0.25–0.60)
Lung-RADS (version 1.1)	0.74 (0.65–0.84)	0.90 (0.50–0.98)	0.49 (0.30–0.64)
Brock score	0.76 (0.62–0.89)	0.91 (0.75–0.99)	0.44 (0.26–0.65)

* Interval between date of sample and date of diagnosis.

**Table 3:** Number of benign nodules, number of lung cancers, the negative predictive value of fDNA <3.15 ng/mL (NPV), and the positive predictive value of fDNA ≥3.15 ng/mL (PPV) for each category of nodule size, Lung-RADS categories, and Brock score.

	cfDNA < 3.15 ng/mL		cfDNA ≥ 3.15 ng/mL		Negative Predictive Value (NPV)	Positive Predictive Value (PPV)
	Benign Nodules (False Positive) *	Lung Cancers (True Positive) *	Benign Nodules (False Positive) *	Lung Cancers (True Positive) *		
Size (mm) of the main nodule						
<6 mm	41	1	19	5	98%	21%
≥6 to <8 mm	11	0	11	3	100%	21%
≥8 to <15 mm	15	3	6	5	83%	45%
≥15 mm	4	2	1	10	67%	91%
Lung-RADS (version 1.1) categories						
1/2	45	2	25	8	96%	24%
3	10	0	7	2	100%	22%
4A	12	2	4	2	86%	33%
4B/4X	4	2	1	11	67%	92%
Brock score						
<1%	38	1	20	5	97%	20%
[1–5%)	20	1	10	2	95%	17%
[5–10%)	4	1	6	1	80%	14%
≥10%	9	3	1	15	75%	94%

* true and false positives, as per the ITALUNG screening protocol.

## Data Availability

Due to restrictions related to patient privacy policies, the data presented in this study are available on request from the corresponding author.
